# Synthetic biology industry: data-driven design is creating new opportunities in biotechnology

**DOI:** 10.1042/ETLS20190040

**Published:** 2019-10-11

**Authors:** Paul S. Freemont

**Affiliations:** 1 Section for Structural and Synthetic Biology, Department of Infectious Disease, Imperial College London, Sir Alexander Fleming Building, South Kensington Campus, London SW7 2AZ, U.K.; 2 UK Innovation and Knowledge Centre for Synthetic Biology SynbiCITE and London BioFoundry, Imperial College Translation & Innovation Hub, White City Campus, 80 Wood Lane, London W12 0BZ, U.K.; 3 UK Dementia Research Institute Care Research and Technology Centre, Imperial College London, Hammersmith Campus, Du Cane Road, London W12 0NN, U.K.

**Keywords:** biotechnology, industry, synthetic biology

## Abstract

Synthetic biology is a rapidly emerging interdisciplinary research field that is primarily built upon foundational advances in molecular biology combined with engineering design. The field considers living systems as programmable at the genetic level and has been defined by the development of new platform technologies. This has spurned a rapid growth in start-up companies and the new synthetic biology industry is growing rapidly, with start-up companies receiving ∼$6.1B investment since 2015 and a global synthetic biology market value estimated to be $14B by 2026. Many of the new start-ups can be grouped within a multi-layer ‘technology stack’. The ‘stack’ comprises a number of technology layers which together can be applied to a diversity of new biotechnology applications like consumer biotechnology products and living therapies. The ‘stack’ also enables new commercial opportunities and value chains similar to the software design and manufacturing revolution of the 20th century. However, the synthetic biology industry is at a crucial point, as it now requires recognisable commercial successes in order for the industry to expand and scale, in terms of investment and companies. However, such expansion may directly challenge the ethos of synthetic biology, in terms of open technology sharing and democratisation, which could unintentionally lead to multi-national corporations and technology monopolies similar to the existing biotechnology/biopharma industry.

## Introduction

Synthetic biology is a rapidly emerging interdisciplinary research field that is built primarily upon foundational advances in molecular biology combined with engineering design. The field considers living systems as programmable at the genetic level and offers the possibility of applying systematic design approaches to constructing new biological systems or cells with human-defined functions [1]. This has led to ideas, such as microbial cell factories for the production and manufacturing of chemicals and materials, smart cells for therapeutics, memory and sensing, and re-engineered plants and algae for energy, vaccines, natural product discovery and efficient food production [2,3]. With the increasing digitisation of DNA and biological data, synthetic biology aims to develop platform/enabling technologies that would allow algorithm-driven biological design, where DNA sequences or parts can be assembled into larger genetic constructs or even genomes or chromosomes. This short perspective is focused on top-down synthetic biology that is driven largely by genetic engineering using genetic parts, although whole genome and chromosome synthesis are gaining rapid utility.

## The synthetic biology design–build–test–learn cycle

**Figure 1.**
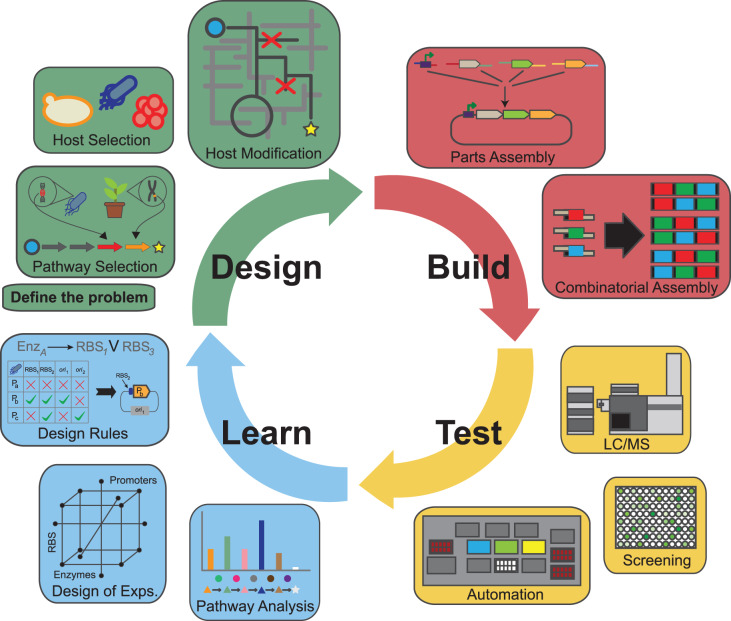
Typical design–build–test–learn cycle diagram from [4]. The cycle begins with Design (D), which involves the computational design of genetic parts, circuits, regulatory and metabolic pathways to whole-genomes; Build (B) involves the physical assembly of those designed genetic components; Test (T) involves the prototyping and testing of the assembled genetic designs; Learn (L) is the application of modelling and computational learning tools, which uses the data obtained in T to inform the design process. Iterations of the DBTL cycle results in genetic designs that aim to fulfil the design specifications established in D. Reproduced from [4] with permission from Dr Chris Petzold.

At the centre of this is data-driven design implemented in the ‘Synthetic Biology Design Cycle’, which encompasses design–build–test–learn processes (Figure 1). The Design and Build modules combine computer-aided design approaches (BioCAD) with DNA assembly followed by testing or prototyping protocols (Test), such that designs can be rapidly screened for their desired specified functions. Tested designs are usually assessed by the measurement of a variety of biological data, primarily ‘omics’ data or specific outputs embedded in the design, such as reporter molecules. These datasets, where appropriate, can be used in computational approaches that include machine learning and statistical Design-of-Experiment, as part of the Learn module. This systematic engineering design approach to constructing living systems perhaps differentiates synthetic biology from more traditional biotechnology or metabolic engineering, as it allows a foundational tool platform based on the synthesis that can be applied to many different application areas [5]. One driving concept is to make ‘biology easier to engineer and more predictive’ and as such the disruptive nature of the technology has been eluded to [6]. Another important philosophical driver of synthetic biology is the concept of sharing and open or pre-competitive technology development, similar to the Open source movement that oversaw the ICT revolution in the 1970s. This has led to the community proposing standardised legal tools to reduce the transaction costs of sharing including the OpenMTA [7].

## Why now? Convergence and BioFABs

Over the last 15–20 years, an attractive agenda around discipline convergence has emerged where interdisciplinary research is encouraged and lauded. This convergence has created opportunities for cross-disciplinary fields to develop in the life sciences like systems biology, computational biology/bioinformatics and chemical biology [8]. Synthetic biology can be considered as a continuation of these trends. A second more profound reason is the availability and decreasing costs of DNA sequencing and DNA synthesis. As synthetic biology considers the genetic code as programmable, the vast array of public available genetic resources from different organisms provides the ‘raw code’ for synthetic biology design considerations. The flipside of DNA sequencing is chemical DNA synthesis and the price of synthetic DNA has significantly reduced, as has the ability to synthesize and assemble larger synthetic DNA constructs [9]. A third driver is the increasing application of automation and software to facilitate synthetic biology research and development, with an ability to potentially screen thousands of genetic parts and designs for expected functions. This has developed into the concept of a Biofoundry originally termed BioFAB [10], which has led to new commercialisation opportunities in tools development. The BioFAB concept has recently been realised in the establishment of a Global Biofoundry Alliance that aims to promote and enable the beneficial use of automation and high-throughput equipment including process scale-up, computer-aided design (CAD) software, and other new workflows and tools in engineering/synthetic biology [11].

## Roadmaps and the bioeconomy

All of these activities have contributed to increasing awareness by policy-makers and governments of the economic potential of synthetic biology and as such, a large number of government-focused strategy documents and/or roadmaps have been published [12]. These include the U.K. governments Biodesign for the Bioeconomy [13]. These reports aim to outline strategies and policies to facilitate the rapid development of a synthetic biology research and technology base, the accelerated translation of academic research and in some cases formal approaches to public dialogues and public engagement. A forward-thinking and technically expansive roadmap has recently been released by the US Engineering Biology Research Consortium of leading universities, industry and research institutes [14]. Road-mapping exercises have occurred against a backdrop of economic projections for synthetic biology, with recent projections converging ∼$14B by 2026 [15]. The current market is dominated by reagents, and tool and contract service companies providing synthetic DNA and molecular biology tool kits for synthetic biology and software. However, as governments grapple with major issues like climate change, and food and energy security, the transition to more sustainable circular economic models like the bioeconomy is becoming very attractive. The bioeconomy can be defined in several ways — simply it describes an economic model where knowledge-based utilisation of biological resources and processes can be applied to the sustainable production and manufacturing of goods, and the provision of services across all economic sectors.

The scale of a bioeconomy model can be impressive. For example, in 2017 the US bio-based economy contributed ∼2% of GDP or $388B after a 20-year sustained period of intense growth [16]. The EU Bioeconomy is estimated to be worth €2.2 trillion, employing 18.6 million people with a major opportunity for growth [17]. The UK Bioeconomy strategy from 2018 shows ∼£220B gross value to the UK economy, taking into account jobs created across the many supply chains and delivery sectors [18]. The need to develop strong circular bioeconomic models will become increasingly important, as carbon taxation and emission levies emerge given the UN countries aspiration of limiting global temperature increases to under 2°C by 2060 [19]. The establishment of a synthetic biology industry in this context is, therefore, extremely timely.

## The synthetic biology technology stack

The CAD approach was developed in the late 1950s and became established in the 1960s, allowing companies cost-benefits in terms of productivity and reproducibility [20]. These early CAD systems evolved into performing engineering calculations (computer-aided engineering, CAE), allowing the design of complex engineered products and extension into manufacturing (computer-aided manufacturing, CAM). Designers use CAD tools to design and simulate their new products before actual manufacturing, and this can be done rapidly and is iteratively often termed ‘rapid prototyping’. For example, new aeroplane designs do not use scale-models for testing in wind tunnels but rely on *in silico* testing before full-scale prototypes are built and tested. This revolution in software-driven design and manufacturing created many of the industries and products we see today. During this revolution, third party software and technology vendors emerged providing solutions for specific parts of the design and manufacturing pipeline, and a whole new software and manufacturing industry emerged creating new value chains and commercial opportunities.

**Figure 2.**
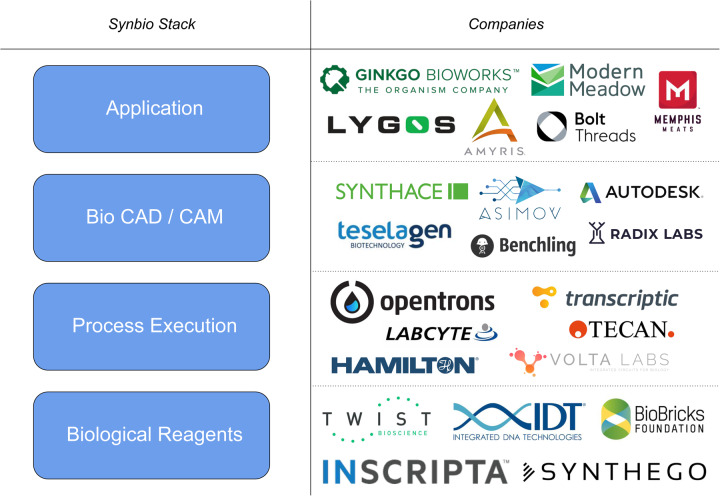
The synthetic biology technology stack. Reproduced from [21] with permission from Will Canine CEO Opentrons. The stack is divided into four layers with the application layer being enabled by the BioCAD design layer that utilises the execution layer to deliver the design/application using biological reagents from the fourth layer. The technology stack is based in part around the DBTL cycle shown in Figure 1.

By analogy, the field of synthetic biology aims to adopt a similar approach to engineering biology. This has led to the synthetic biology technology ‘stack’ based on the engineering concepts of abstraction, hierarchy and modularity, used to overcome the complexity of engineering biological systems [6]. This approach allows highly complex systems to be broken into manageable layers/modules and components, exemplified by software engineering, where modules are sub-routines. The advantage of modularity is that each layer is manageable in terms of complexity. The synthetic biology technology stack has been proposed to comprise four layers [21] (Figure 2); an application layer with defined products; a BioCAD/CAM software layer for design; a process execution layer that would involve automation and a biological reagents layer where components or parts are combined based on the design specification. Interestingly, this technology stack is now being enabled by emerging synthetic biology start-up companies that offer ‘off-the-shelf’ solutions in each of the abstraction layers (Figure 2). The novelty of the synthetic biology approach to biotechnology is illustrated by this approach, and no doubt further abstraction layers enabled by new synthetic biology companies will be developed, that identify commercial niches and opportunities within this framework. The synthetic biology technology stack also allows new commercial value chains to be developed, providing opportunities for Business-to-Business transactions within and between companies in the stack.

## Synthetic biology industry: applications and sectors

As the adoption of the synthetic biology technology stack continues, new commercial applications for synthetic biology are being realised beyond the usual applications involving industrial biotechnology or metabolic engineering. The first area is consumer biotechnology, which has seen rapid growth, exemplified by companies like Bolt Threads, Spiber and Modern Meadow. These companies focus on producing synthetic yet ‘natural’ biomaterials that are sustainable and do not require animals or spiders/worms. These new biomaterial-based products are being marketed as sustainable fashion accessories and/or incorporated into existing clothing products. Other companies, such as Colorifix are developing new dying technologies with materials dyed by engineered bacteria. These exemplars are part of a growing Biodesign movement [22], where artists, designers, scientists and engineers explore different collaboration models for applying biotechnology to different application areas now and in the future. An innovative example is Opencell, which provides affordable shipping container laboratories for new biotechnology biodesign start-ups [23]. The growing area of Biodesign innovation illustrates the new intersections of synthetic biology, which is extending into other consumer biotechnology products like, pet food (Wild Earth) and cosmetics and fragrances (Ginko Bioworks). The second rapidly growing application area is ‘living medicines’. These involve the engineering of living cells, including bacteria, to perform therapeutic functions inside or on the surface of the body. Exemplars range from products like Synthetic Biotic^TM^ (Synlogic), designed to treat inflammatory bowel disease, or start-up companies like Senti Bioscience, who aim to use gene circuit designs to engineer a new generation of cell therapies. Another example is the start-up Azitra, who are engineering the skin microbiome to treat a rare but serious skin condition called Netherton syndrome. This sector is growing rapidly and is building upon the earlier successes of CAR-T cell cancer therapies. There are many examples of companies in this area, although considerations like manufacturing costs and FDA/regulatory approval may inhibit some short-term commercial successes.

With these exciting new application areas, issues around societal acceptance, governance and adoption have arisen, which are often framed around Responsible Research and Innovation or RRI [24]. In this context, a question that new synthetic biology companies might need to consider is what can synthetic biology industry do for society? Whilst many synthetic biology companies are increasingly becoming aware of these issues, the implementation of any company policies or governance structure to address them is limited and often compromised through legal governance structures that date back to the mid-19th century. The new emerging synthetic biology industry has a real opportunity to tackle some of the most difficult global problems that society has ever faced, whilst taking advantage of exciting new circular economic models and green investment around the bioeconomy. Whether the new synthetic biology industry will take up these major societal challenges is still an open question.

## Synthetic biology industry: the future

In this short perspective, I have described how synthetic biology is a platform design technology that is leading to the establishment of a technology stack, serviced and developed by many new start-up companies. The synthetic biology technology stack is being applied in different application sectors, which differentiates synthetic biology from the more traditional industrial biotechnology or pharmaceutical industry. As the synthetic biology industry develops, realities will emerge with companies needing to satisfy investor demands to become financially sustainable and commercially successful. Although current investment in new start-ups is growing rapidly, with ∼$6.1B investment since 2015 [22], the synthetic biology industry is still at a nascent stage and requires some of the major new companies like Ginko Bioworks, Zymergen and Amyris to become commercially successful. However, such commercial forces may lead to the development of behemoth synthetic biology corporations that resemble existing multi-nationals, thus inadvertently creating technology monopolies. The ethos of synthetic biology as a disruptive and societally enabling technology could thus be lost with existing commercialisation models resulting in ‘business as usual’ — although compromises will need to be met to ensure that the industry thrives and the technology is exploited to its full potential for societal benefit. However, a new generation of synthetic biology entrepreneurs, academic, start-ups and investors see significant advantages in pre-competitive open technology platforms. By creating an open technology environment, it is envisaged that many more new start-ups can be formed in new applications, areas and market sectors. As John F. Kennedy once said, ‘The rising tide lifts all boats’. There are parts of the synthetic biology start-up community that are committed to sharing open technology developments that can be used to ‘change the world’ for good — at least for now.

## Summary

- The new synthetic biology industry is growing rapidly, driven primarily by new start-up companies that have received ∼$6.1B investment since 2015 with the market value estimated to be $14B by 2026.
- The synthetic biology industry can be segmented around a ‘Synthetic Biology Technology Stack’ comprising four abstraction layers of BioCAD design, experimental execution, biological reagents and applications.
- New applications and product areas are being identified with a growing number of companies involved in consumer biotechnology products and living therapeutics.
- The ethos of the synthetic biology field, in terms of open technology sharing and democratisation, may be challenged by financial and market realities that result in synthetic biology corporations and technology monopolies.

## Abbreviation

CAD: computer-aided design
CAE: computer-aided engineering
CAM: computer-aided manufacturing
